# Triboelectric Spectroscopy for In Situ Chemical Analysis of Liquids

**DOI:** 10.1021/jacs.3c13674

**Published:** 2024-02-07

**Authors:** Jinyang Zhang, Xuejiao Wang, Long Zhang, Shiquan Lin, Simone Ciampi, Zhong Lin Wang

**Affiliations:** † Beijing Institute of Nanoenergy and Nanosystems, Chinese Academy of Sciences, Beijing 100083, P. R. China; ‡ School of Nanoscience and Technology, University of Chinese Academy of Sciences, Beijing 100049, P. R. China; § Center on Nanoenergy Research, School of Physical Science and Technology, Guangxi University, Nanning, Guangxi 530004, P. R. China; ∥ Institute of Quantum and Sustainable Technology (IQST), School of Chemistry and Chemical Engineering, Jiangsu University, Zhenjiang 212013, China; ⊥ School of Molecular and Life Sciences, Curtin University, Bentley, Western, Australia 6102, Australia; # Yonsei Frontier Lab, Yonsei University, Seoul 03722, Republic of Korea; ¶ School of Materials Science and Engineering, Georgia Institute of Technology, Atlanta, Georgia 30332-0245, United States

## Abstract

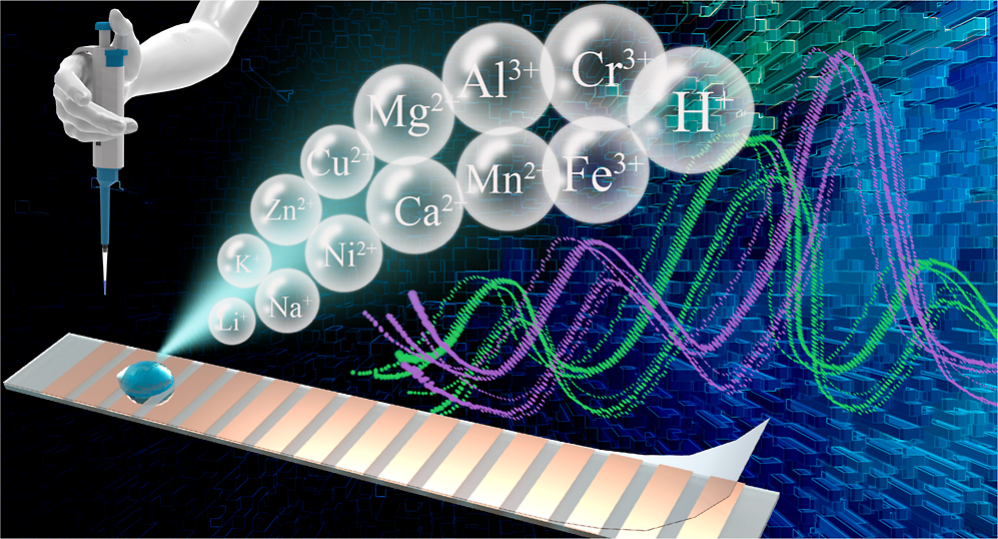

Chemical analysis of ions and small organic molecules in liquid samples is crucial for applications in chemistry, biology, environmental sciences, and health monitoring. Mainstream electrochemical and chromatographic techniques often suffer from complex and lengthy sample preparation and testing procedures and require either bulky or expensive instrumentation. Here, we combine triboelectrification and charge transfer on the surface of electrical insulators to demonstrate the concept of triboelectric spectroscopy (TES) for chemical analysis. As a drop of the liquid sample slides along an insulating reclined plane, the local triboelectrification of the surface is recorded, and the charge pattern along the sample trajectory is used to build a fingerprinting of the charge transfer spectroscopy. Chemical information extracted from the charge transfer pattern enables a new nondestructive and ultrafast (<1 s) tool for chemical analysis. TES profiles are unique, and through an automated identification, it is possible to match against standard and hence detect over 30 types of common salts, acids, bases and organic molecules. The qualitative and quantitative accuracies of the TES methodology is close to 93%, and the detection limit is as low as ppb levels. Instruments for TES chemical analysis are portable and can be further miniaturized, opening a path to in situ and rapid chemical detection relying on inexpensive, portable low-tech instrumentation.

## Introduction

Chemical analysis is central to all fields of science, technology, engineering, and health.^1−4^ Strategies for the analysis of ions and organics include optical,^5^ electrochemical,^6^ and chromatographic methods.^7^ Optical methods often lack selectivity and generally require large and expensive instrumentation,^8^ and mainstream chromatographic instruments remain far from portable and inexpensive.^9^ Electrochemical methods fail to resolve analytes of similar redox potential, and quantitative analysis is often complicated by mass transport and kinetic factors at the electrode.^10^

Electrification and charge exchange at interfaces are however not limited to electrical conductors and semiconductors:^11,12^ Electrical insulators can gain a static charge and guide redox chemistry in response to mechanical forces.^13,14^ This phenomenon—triboelectricity—is not restricted to insulators experiencing large mechanic forces, and even the gentle sliding of a rain droplet yields a measurable surface charge on a plastic surface.^15−17^ Triboelectrification between a liquid and solid involves electron transfer, ion movement, and formation of an electric double layer.^18^ For example, when a water droplet slides on the titled fluorinated ethylene propylene (FEP) surface, the electrons will first transfer from water molecules to FEP, resulting in the negatively charged FEP surface and positively charged liquid droplet. The cations in the liquid will further adsorb on the negatively charged FEP interface due to electrostatic force. Thus, after the droplet moves away, some adsorbed cations should stay on the FEP surface. Therefore, the net charge on solid being measured by, for instance, an electrometer, is the difference between the electron transfer and ion adsorption events.^19,20^ In this way, the type of ions/groups should have different adsorption on the charged dielectric surface due to the difference in mass, radius, and charge and therefore will affect the net charge measured at the liquid–solid interface. Since triboelectrification and charge transfer at “sliding” liquid–solid interfaces are known to depend on the chemical nature of the water droplet,^19,21,22^ there is the possibility of harnessing toward analytical applications.

Notably, mapping the charge transferred along the trajectory of a sliding droplet previously showed that the triboelectrification of the liquid–solid interface is not uniform and depends on the chemical properties of the solution.^22^ We postulated therefore the possibility of detecting chemicals by analyzing the spectral tuning of the charge transfer spectroscopy on the movement trajectory of the droplet. We term this concept triboelectric spectroscopy (TES) for chemical analysis. In the following, we describe the scope of TES for the nondestructive analysis of a wide range of both inorganic and organic chemicals.

## Experimental Section

### Materials

Redistilled solvents and Milli-Q water (>18 MΩ cm) were used for substrate cleaning and preparation of solutions. An FEP (30 μm, DAIKIN) film was used for triboelectrification with liquid droplets. Sodium chloride (99.5%), sodium iodide (99.5%), sodium nitrate (99%), sodium bicarbonate (99.5%), sodium carbonate (99.5%), sodium sulfite (98%), sodium sulfate (99%), magnesium sulfate (99%), potassium chloride (99.5%), potassium ferricyanide (99.5%), calcium chloride (97%), chromic nitrate (99%), manganese(II) chloride (99%), manganese(II) sulfate (99%), ferric nitrate (98.5%), nickel(II) chloride (99%), copper sulfate (99%), copper(II) nitrate (99%), zinc nitrate (99%), zinc acetate (98%), hydrochloric acid (37%), sulfuric acid (98%), nitric acid (68%), potassium hydroxide (99.9%), and sodium hydroxide (97%) were purchased from Macklin. Ethanol (99.7%) and acetone (99.5%) were obtained from Yong Da Chemical.

### Fabrication of Experimental Setup

The copper electrode array was composed of 16 individual electrodes with a width of 1.5 cm and length of 10 cm and on a smooth and clean poly(methyl methacrylate) (PMMA) plate (38.5 × 10 × 0.3 cm^3^); the PMMA plates were used as the backboards. The distance between the electrodes is 0.5 cm. The FEP film was carefully attached to the PMMA plate. FEP film was extensively washed with Milli-Q water and ethanol prior to each experiment to remove static charges and residual ions on the FEP surface.

### Electrical Measurement

The copper electrode array on the PMMA plate was connected to National Instruments NI PXle-8880 electrometer with multiple channels. The NI PXle-8880 electrometer with a Labview program on a computer was used to measure the electric signal on each copper electrode produced by the interaction between the droplet and the FEP. In all experiments, the volume of liquid droplet is 30 μL per drop, the droplet height was set as 1 cm, the air humidity was 20%, and the temperature was 20 °C. The liquid droplet was dripped by a syringe pump (LEADFLUID, TYD01–01).

## Results and Discussion

**Figure 1:**
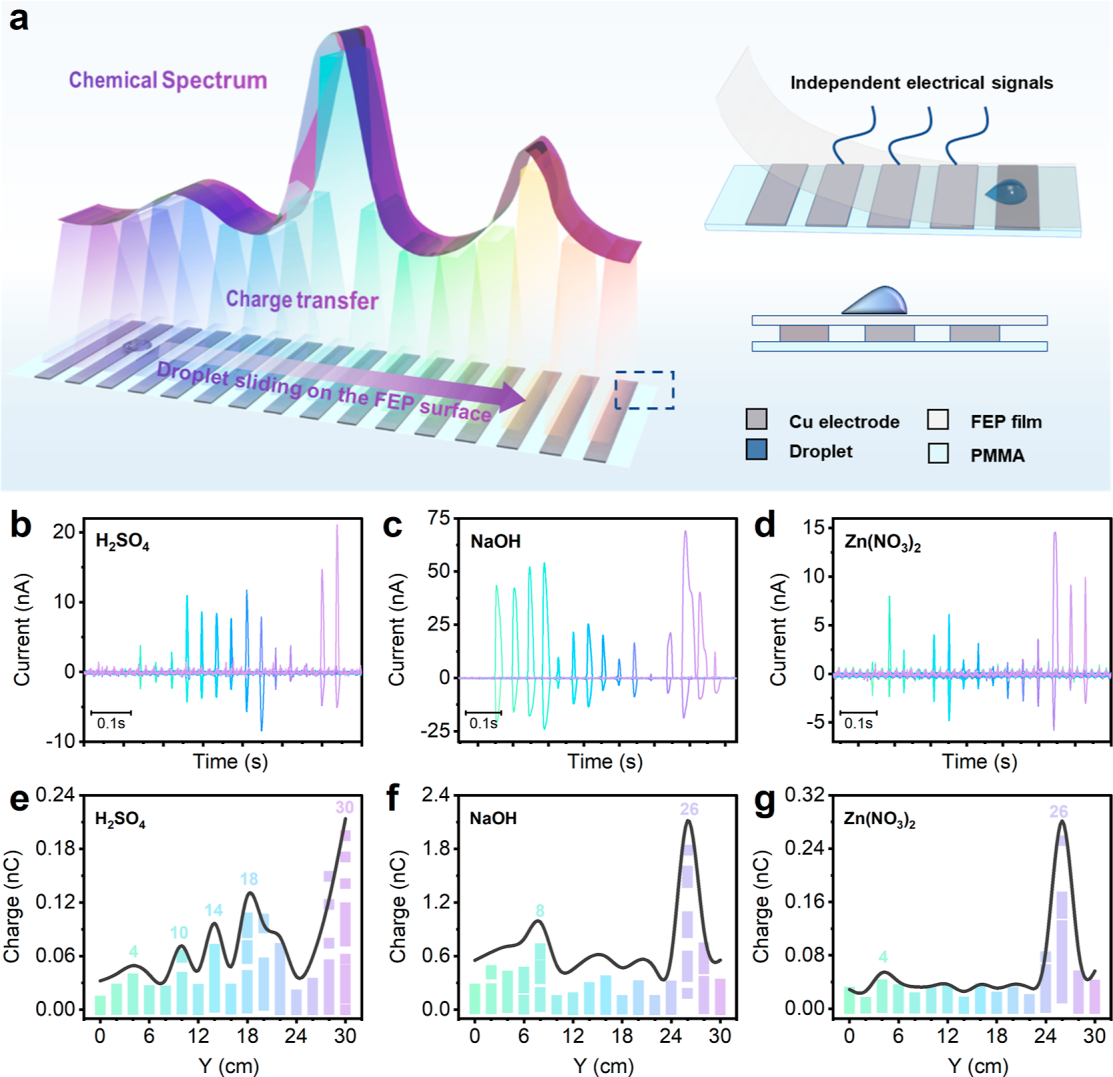
TES for chemical analysis. (a) Experimental design based on the movement of a liquid droplet along a dielectric surface separating a sample from an array of copper band electrodes. The electrodes rest on a PMMA support, and the dielectric layer is a FEP film. As the droplet moves along the FEP surface, the charge measured by each electrode is recorded by an electrometer and used to build a TES profile. (b–d) Typical current output profile when the droplets with different chemicals [H_2_SO_4_ (pH 3), NaOH (pH 13), and Zn(NO_3_)_2_ (1 M)] slide across the FEP film. (e–g) TES data recorded for H_2_SO_4_ (e), NaOH (f), and Zn(NO_3_)_2_ (g) samples. Number in *y* axis represents the sliding distance (the position of the first electrode is 0). Each curve is the mean of 20 independent experiments, and the small squares below the curve are the data of 20 independent experiments.

Figure 1a shows the experimental design of triboelectrification-induced charge transfer spectroscopy. The equipment consists of three layers: a PMMA plate as the bottom layer for support, an FEP film top layer for the triboelectrification of the liquid sample droplet, and an array of copper electrodes, placed between the FEP film and the PMMA plate, for the electrostatic induction. FEP was chosen due to its high water contact angle, which allows the droplets to slide smoothly off its surface, and strong electron affinity (higher charge transfer).^16^ The distance between electrodes is 0.5 cm to reduce the interference between the electrodes. When a liquid droplet (∼30 μL per drop needed) was released from a grounded stainless-steel needle by a syringe pump at a fixed height (∼1 cm above the FEP surface), sliding across on the tilted FEP surface (tilted angle is 50°), the transferred charges at different sliding times, *Q*_(*t*)_, were induced and detected through the Cu electrode array at the same time (Supporting Information, Figure S1). Figure 1b–d shows typical current output profiles when the droplet containing different chemicals slid across the FEP surface. Taking for example, the case of H_2_SO_4_, NaOH, and Zn(NO_3_)_2_, the detection time (total time for the droplet to slide across the FEP film) is less than 1 s. The working mechanism of charge induced at each electrode is shown in Supporting Information, Figure S2. Based on the obtained current curves, the transferred charge at each electrode can be calculated. The detailed calculation of the corresponding transferred charges at each electrode is shown in Supporting Information, Figure S3. Therefore, according to the transferred charge at each electrode, we plotted the triboelectrification-induced charge transfer spectroscopy in Figure 1e–g and Figure S4. It is obvious that the characteristic TES for droplets containing acid (H_2_SO_4_, pH 3), base (NaOH, pH 13), and salts (Zn(NO_3_)_2_, 1 M) exhibits distinct features and can be differentiated from the following aspects: (1) peaks positions: for example, in the case of H_2_SO_4_, there are five peaks located at 4, 10, 14, 18, and 30 cm, separately, whereas for NaOH, only two peaks are located at 8 and 26 cm; (2) peak-to-peak ratio: for instance, the peak ratios for NaOH and Zn(NO_3_)_2_ are 0.34 and 0.11, respectively; and (3) amount of transferred charges at the maximum peak: the maximum peaks for H_2_SO_4_, NaOH, and Zn(NO_3_)_2_ corresponding to transferred charges are 0.07, 0.86, and 0.09 nC, respectively.

**Figure 2:**
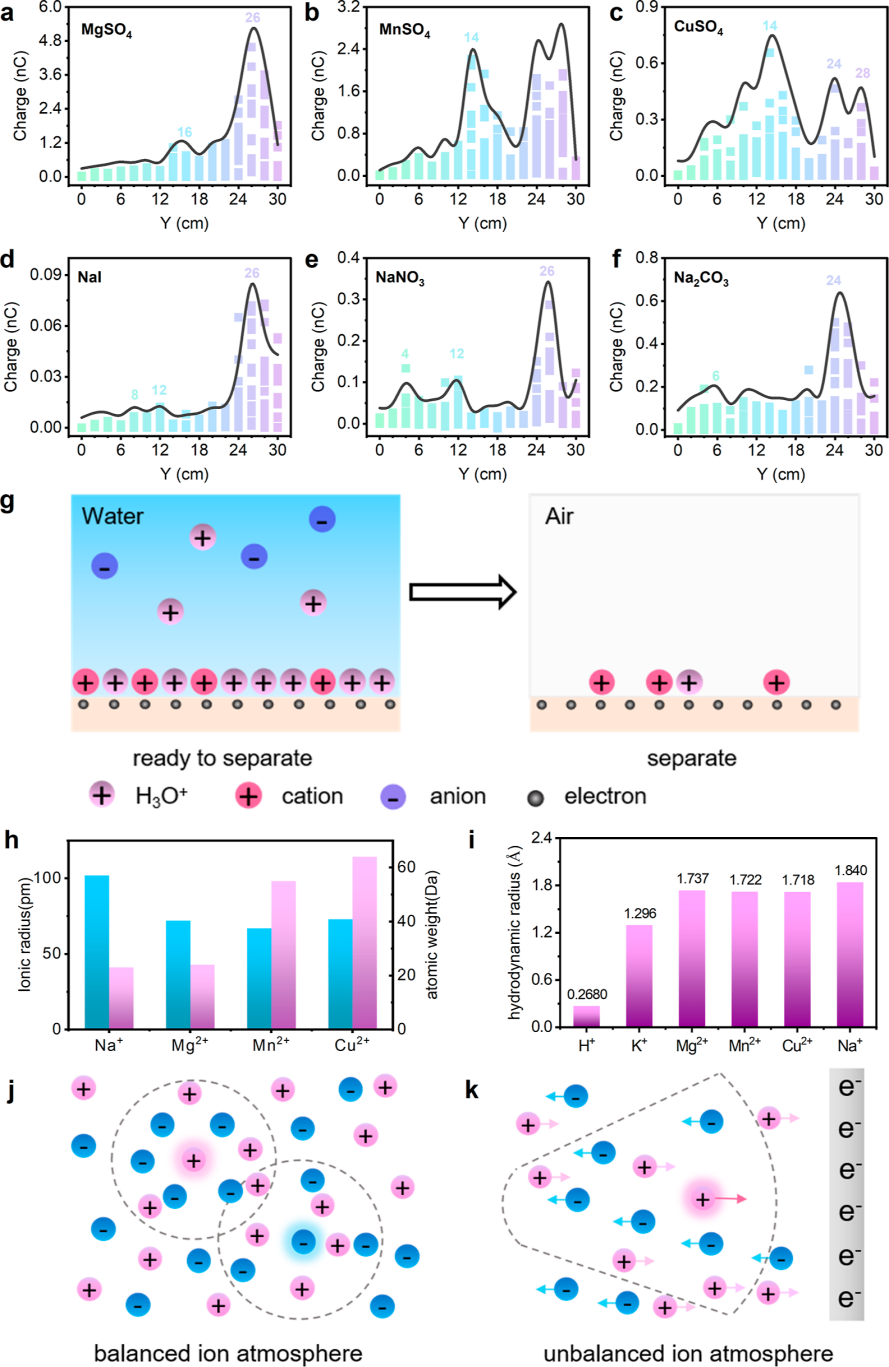
Role of cations and anions in liquid droplets on the characteristic TES. (a–c) TES recorded for 1 M MgSO_4_, MnSO_4_, and CuSO_4_ with same anions. (d–f) TES recorded for 1 M NaI, NaNO_3_, and Na_2_CO_3_ with same cations. (g) Schematic illustration of cations’ competitive adsorption and desorption on FEP surface. (h) Relation between the ionic radius and atomic weight of cations, including Na^+^, Mg^2+^, Mn^2+^, and Cu^2+^, to the characteristic TES. (i) Hydrodynamic radius for various ions. (j–k) Ion atmosphere in bulk solution (j) and on a negatively charged solid interface (k).

Triboelectrification between liquid and solid is different with solid–solid contact. When a liquid droplet establishes contact with a clean FEP surface, the electrons will first transfer from water molecules to FEP, resulting in the negatively charged FEP surface and positively charged liquid droplet (H_3_O^+^ generated at interface).^21,22^ The cations in the liquid droplet are further adsorbed on the negatively charged FEP interface.^18^ Thus, the cation dynamic should be accounted for the characteristic TES,^23^ and our experiments further validated this in Figure 2a–c. The spectroscopic findings recorded for MgSO_4_, MnSO_4_, and CuSO_4_ with the same anions can be clearly distinguished from their peak positions, peak-to-peak ratios, and transferred charges at the maximum peak. The characteristic TES curves recorded for chemicals with same cations, for example, NaI, NaNO_3_, and Na_2_CO_3_, in Figure 2d–f show that anions in the liquid droplet are also affecting the TES but not significantly. This should be due to the negatively charged FEP absorbing cations in water and thus the higher selectivity to cations than that to anions. The droplet movement possibly affects the charge pattern on the FEP surface. To test this hypothesis, we have tried to observe the contact angles (Supporting Information, Figures S5 and S6) of droplets containing various chemicals. However, contrary to our intuition, the contact angles for the chemicals, as shown in Supporting Information, Figure S5, are found to be similar. The snapshots in Supporting Information, Figure S7, also indicate that the motion of droplets containing various chemicals on the FEP film looks almost the same. The findings presented above suggest that droplet movement is not the dominant factor in terms of shifting of the TES peaks. Interfacial charge transfer between liquid and solid is related to the process of both electron transfer and ion adsorption,^20^ thus the net charges detected by electrometer at different sliding times can be expressed as
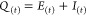
where *E*_(*t*)_ and *I*_(t)_ denote the amounts of electrons transfer and ions adsorbed at different sliding times, respectively. However, the process of cation adsorption and desorption on the negatively charged FEP surface should be related to *Q*_(*t*)_. Thus, we proposed that the various charge transfer spectroscopy findings for ions could be due to the difference in the amount of cations adsorbed on the FEP surface as well as the amount of the cations carried away by the droplet as it slides away (Figure 2g). In fact, the ionic radius of Na^+^ is significantly larger than that of Mg^2+^, Mn^2+^, and Cu^2+^, and the atomic weight of Na^+^ is the smallest compared with that of Mg^2+^, Mn^2+^, and Cu^2+^ (Figure 2h), and the onset of the charge peak in liquid containing Na^+^ is the fastest we have observed (Figure 2a–f). Data in Supporting Information, Figure S8 and Table S1 also prove this: H^+^ (such as HCl, H_2_SO_4_, and HNO_3_) and K^+^ (such as KCl and KOH) have larger ionic radius and smaller atomic weight, and the onset of the charge peak is the fastest observed. It is easy to understand that the lighter the cation is, the faster it should adsorb on the charged surface. For larger ionic radius, we have considered the possibility of hydrodynamic radius affecting TES. In fact, the hydrodynamic radius of H^+^ (0.268 Å) and K^+^ (1.296 Å) is significantly smaller compared with those of Mg^2+^ (1.737 Å),^24^ Mn^2+^ (1.722 Å), and Cu^2+^ (1.718 Å) in Figure 2i,^24^ and the onset of the charge peaks for H^+^ and K^+^ are the fastest observed. However, the situation in Na^+^ appears to be more complicated: Na^+^ has a hydrodynamic radius similar to that of Mg^2+^ (Figure 2i), but the onset of the charge peak in liquid containing Na^+^ is faster than we have observed.

We also note that the cation dynamic alone cannot explain the charge pattern: liquid droplets with the same cation and different anions have different TES findings (Figure 2d–f). The ion atmosphere in bulk solution is different from the ion atmosphere on charged solid interface,^25^ which is shown in Figure 2j–k. Due to the electrostatic attraction between anions and cations, there is a spherically symmetrical negative charge atmosphere around the cation, which is called “ion atmosphere”. That is to say, in an electrolyte solution, each ion is surrounded by an ion atmosphere with opposite charges, which can be called a balanced ion atmosphere. However, the situation is different when the negative charges on the liquid–solid interface are applied (Figure 2k).^26^ In this case, the ion atmosphere and thus the anions should affect the migration rate of the central cation to the negatively charged FEP surface. Figure 2k shows that when the central cation migrates to the negatively charged FEP interface, the anions in the ion atmosphere migrate in the opposite direction at the same time and leave the ion atmosphere, while the ions close to the FEP surface continue to enter the ion atmosphere, leading to the continuous destruction and regeneration of the ion atmosphere. This process probably will hinder the migration of the central cation to negatively charged FEP surface, hence affecting the charge transfer distribution and TES. In fact, we found that anions with relatively larger ionic radius led to the slower onset of the charge peak. For example, I^–^ (220 pm) and SO_4_^2–^ (244 pm) have larger ionic radius compared with that of NO_3_^–^ (177 pm) and CO_3_^2–^ (164 pm), and the onset of the charge peak is slower (Figure 2a–f).

**Figure 3:**
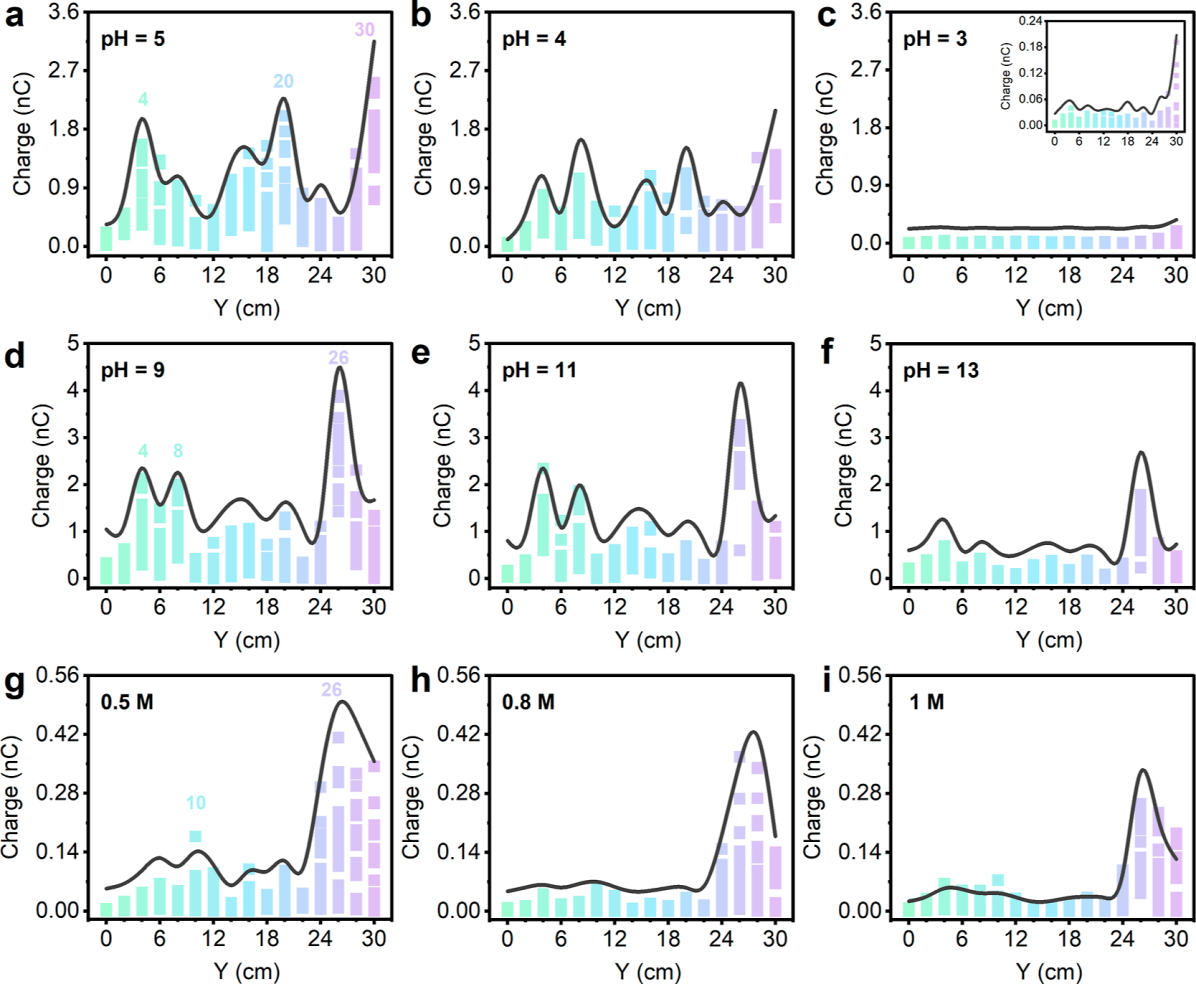
Performance of characteristic TES curve for pH and ion concentration detection. (a–c) Spectroscopy recorded for HCl with various pH values. (d–f) Spectroscopy recorded for KOH with various pH values. (g–i) Spectroscopy recorded for K_3_Fe(CN)_6_ with different concentrations, 0.5, 0.8, and 1 M, respectively. Each curve is derived from the mean of 20 independent experiments, and the small squares below the curve are the data of 20 independent experiments.

Modern chemical analysis requires the ability to determine concentrations at the same time. Thus, in order to evaluate the performance of characteristic TES curves for pH and ion concentration, we have conducted experiments on various pH values of HCl and KOH, along with different concentrations of K_3_Fe(CN)_6_ in Figure 3. When characteristic TES curves for samples of different pH are compared, what becomes apparent is that the transferred charges at each peak in characteristic TES curves scale with their pH magnitude (Figure 3a–f). For example, when pH value of HCl decreases from 5 to 3, the transferred charge at each peak decreases almost linearly with the liquid concentration (Figure 3a–c), that is, lower concentration of ions in water leads to the increase of total transferred charges, and this is consistent with the results of our previous work.^16,21^ Data in Figure 3d–f also support this: increasing the pH value of KOH leads to a decrease of transferred charge at each peak. However, the shape of the characteristic TES curve, including the peaks’ positions and peak-to-peak ratio, is almost similar. When considering the concentration effect on the characteristic TES curves, the situation is similar compared with that of acid and base: when the concentration of K_3_Fe(CN)_6_ increases from 0.5 to 1 M, the total transferred charges decreases, but the shape of characteristic TES curve keeps. This property further reinforces our characteristic TES being mediated by triboelectrification at liquid–solid interface is unique in distinguishing ions in water and at the same time can analyze the pH and concentration. Further, we have also tried to test a lower concentration of Fe(NO_3_)_3_ in water at ppb level. The results in Supporting Information, Figure S9, show that TES is also suitable for the detection of trace analytes in water, and the detection limit can reach the ppb level at the least.

The chemical analysis database with automated identification program, including common salts, acids, bases, and organic solutions, has also been built to help identify chemicals systematically and fast, as shown in Supporting Information, Figure S10. As long as we enter the peaks positions and the corresponding transferred charges into the database search menu, one is able to identify what the analyzed ion is. Further, we have performed randomized experiments for common chemicals in our database (the number of measurements is 80) and tapped the peak values and corresponding charges into the database search menu, and we found that the qualitative accuracy of our TES is more than 93%, while quantitative accuracy is also as high as 93%. The environmental factors, including temperature and humidity, are known to affect the charge transfer at interfaces.^27,28^ Further, in order to evaluate the measuring environment of our characteristic TES, the experiments at different temperatures and humidities are performed, as shown in Supporting Information, Figure S11. When the temperature increases from 15 to 30°, the shape of characteristic TES curve of 1 M Cu(NO_3_)_2_ is almost unchanged (Supporting Information, Figure S11a–c). This indicates that our characteristic TES curve is unaffected and reliable under normal temperatures (15 –30°). The humidity effect is also studied, as shown in Supporting Information, Figure S11b,d,e, and when the humidity increases from 20 to 40%, the shape of characteristic TES curve remains unchanged, but the amount of charge transfer at the maximum peak increases from 0.015 to 0.038 nC, which shows that the measuring environment of our characteristic TES curve has relatively high requirements for humidity control when the quantitative analysis is needed.

**Figure 4:**
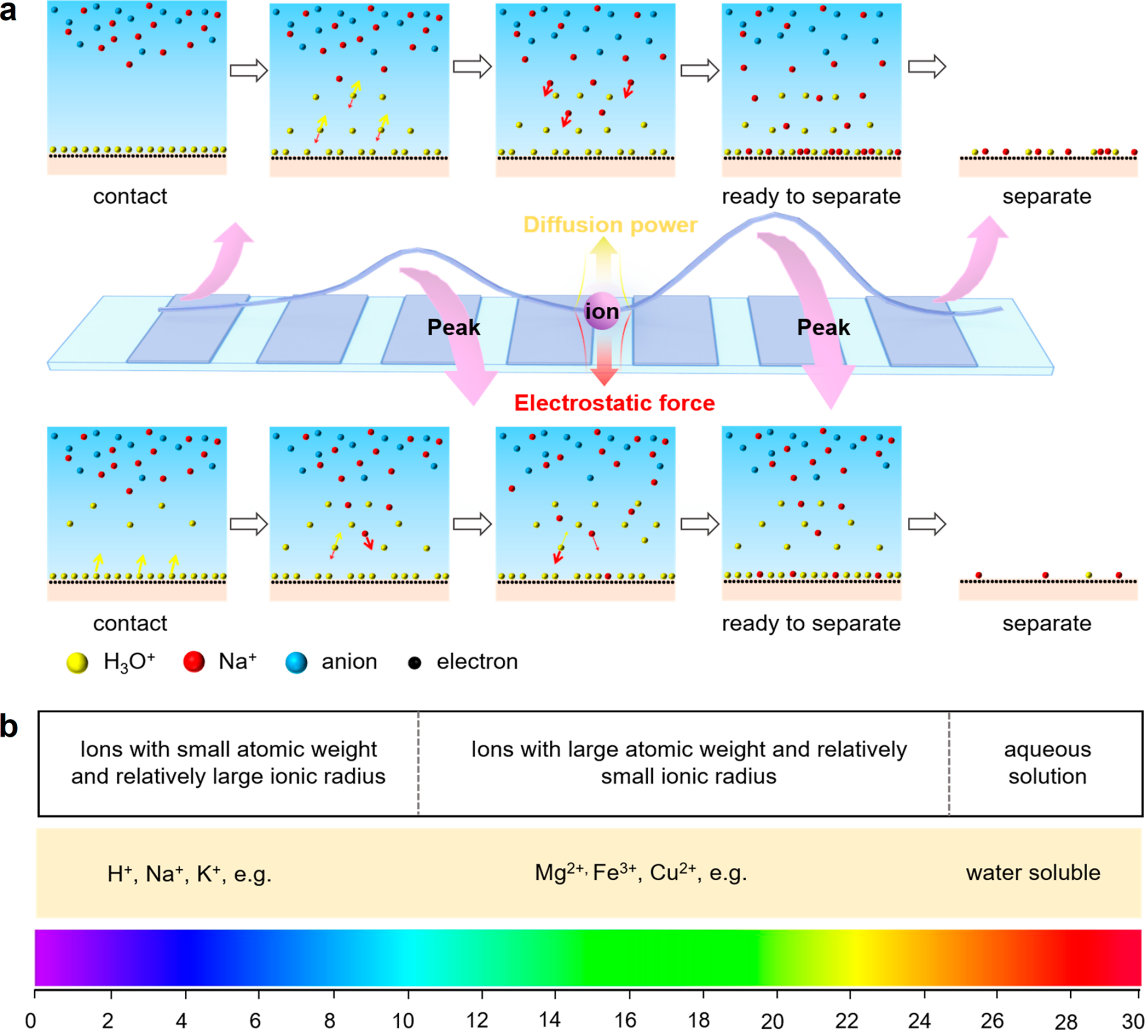
Mechanism of generation of characteristic TES curve. (a) Schematic illustration of the working principle of TES for chemical analysis. (b) TES and corresponding ions.

The scope of TES for the nondestructive analysis of a wide range of chemicals has been discussed above, but it is important to understand why triboelectrification gives different chemical components their own signature spectra. Based on our observations, we proposed that the mechanism of generation of characteristic TES curves for various chemicals is due to the competition of cations and interfacial concentration distribution of H_3_O^+^. For example, when a fresh liquid droplet containing Na^+^ comes into contact with a clean FEP film at the very beginning, the electron will transfer from water molecules to FEP first, resulting in the negatively charged FEP surface and the highest concentration of H_3_O^+^ (H_2_O^+^ + H_2_O = H_3_O^+^) generated at the liquid–solid interface (Figure 4a). In this way, a large amount of H_3_O^+^ begins to diffuse into the solution due to the larger concentration gradient of H_3_O^+^ at the interface. After that, a large number of Na^+^ molecules move to the FEP surface to replace H_3_O^+^ due to thermal motion and electrostatic attraction. When the droplet begins to separate from the FEP surface with Cu electrode under it, some of adsorbed H_3_O^+^ and Na^+^ will be taken away by the moving droplet, and some remain on the solid surface (Figure 4a). Therefore, the transferred charge we measured is the difference between the amount of electron transfer and the remaining ion adsorption. The previous experiments prove that the charge transfer at water/FEP surface is the largest,^16,21^ which shows that H_3_O^+^ is the easily taken away by the moving droplet. Thus, Na^+^ stays more easily on the solid surface than H_3_O^+^, causing the surface electrons to be sufficiently shielded by the adsorbed Na^+^. This is probably the reason the peak was not detected at the beginning for all chemicals (position 1 in the Supporting Information, Figure S12a). As the droplet continues to slide and triboelectrify with the FEP surface, the concentration of H_3_O^+^ in the droplet surface gradually increases, resulting in the continuous decrease of the concentration gradient of H_3_O^+^ at the interface. Thus, the diffusion of H_3_O^+^ at the interface gradually decreases, and less H_3_O^+^ will leave the solid surface. In this situation, H_3_O^+^ and Na^+^ will compete for adsorption on the charged FEP surface due to electrostatic attraction. The adsorption of Na^+^ on the charged solid surface is related to the valence state, weight, radius, concentration, and electric field strength. While, for H_3_O^+^, it is only related to concentration gradient and electric field strength. When the electrostatic force is larger than the diffusion force to H_3_O^+^, more H_3_O^+^ is prone to be adsorbed on the FEP. Thus, the electrons will be shielded, leading to the decreased electric field strength and less Na^+^ being adsorbed. Until one point, the adsorption of H_3_O^+^ on the interface increased and reached a peak in an instant. When the droplet leaves, the transfer charge increases sharply to a peak due to the large number of H_3_O^+^ that is easily taken away by the moving droplet (electrons are barely shielded). This is probably the reason the peak appears (position 2 in the Supporting Information, Figure S12a). After the peak, the H_3_O^+^ at the interface cannot be replenished in a short time, thus the concentration of H_3_O^+^ at droplet boundary is higher than that at the FEP surface. In this way, H_3_O^+^ starts to move toward the FEP, and the concentration of H_3_O^+^ at the interface begins to return to the initial state (position 3 in Supporting Information, Figure S12a), and another cycle begins. Therefore, the peak discontinuity appears with the continuous change of the interfacial concentration distribution of H_3_O^+^ at the interface (Supporting Information, Figure S13). In this way, the difference in weight, radius, and charge of ions leads to the difference in the extent of ion adsorption and desorption on the charged FEP surface under the same electric field strength and therefore will affect the periodic adsorption process and concentration distribution of H_3_O^+^ at the liquid–solid interface. This should be the reason for the various peak positions and peak values for droplets containing different types of ions, giving the TES ion discrimination capability.

The onset of the first peak in the liquid containing cations with larger ionic radius and lighter atomic weight is the fastest observed (Figure 4b), for instance, the chemicals that contain Na^+^ and K^+^ (Figure 4b and Supporting Information, Table S1). The reason should be ascribed to the faster moving of cations under an electric field that could accelerate the periodic adsorption process of H_3_O^+^ at the interface. Therefore, for cations with larger ionic radius (weak electrostatic attraction with anions) and lighter atomic weight, the movement speed is faster under the applied negative electric field, thus the shorter time required to disrupt the diffusion process of H_3_O^+^ (Supporting Information, Figure S13). It is noted that there is always a peak around 26–30 when aqueous solutions or organic solutions that can dissolve in water (for example, ethanol and acetone) are applied in the experiment (Supporting Information, Table S1); we therefore proposed that this may be a characteristic peak of H^+^, and the stronger the acidity, the more the peak shifts to the right. In fact, the peaks for HNO_3_, HCl, and H_2_SO_4_ are all located at 30 (see database in Supporting Information, Table S1). Further, we have also evaluated the performance of characteristic TES on a real mix sample. When a real tap water droplet slides through the FEP surface, the spectroscopy shows a high probability of Ca^2+^, Na^+^, and Mg^2+^ in the sample compared to that in our database, which is consistent with ICP measurement in Supporting Information, Figure S14. We also tried to use 26 cu electrodes (Figure S15). Compared with a 15 mm width electrode, a 7 mm electrode reduces the overall charge transfer, but the peak position in the obtained spectroscopy is almost unchanged.

## Conclusions

In this work, we have established a real-time triboelectric spectroscopy (TES) for chemical analysis that allows ultrafast (less than 1 s) and precise analysis of a wide range of ions in water. The approach relies on triboelectrification at the liquid–solid interface, and the form of the spectral tuning of a charge transfer pattern allows in situ detection of the chemicals in liquid. We successfully obtained the characteristic TES of more than 30 types of common salts, acids, bases, and organic solutions, and the chemical analysis database with automated identification program is also built to help identify chemicals systematically and fast. The qualitative and quantitative accuracies of the TES methodology is close to 93%, and the detection limit is as low as ppb levels. The mechanism of generation of characteristic TES for various chemicals is discussed in detail, and we proposed that the root cause behind the peaks in the characteristic TES is the competition of cation adsorption on the charged FEP surface and interfacial concentration distribution of H_3_O^+^. Our method is applicable to the most common chemicals, including water-soluble salts, acids, bases, and organic solvents (database in Supporting Information, Table S1). Strict and complicated sample preparation is not required. Another advantage of sensing of the chemicals by characteristic TES is that this method could in principle realize the ultralight, portable and wireless requirements of modern chemical analysis through integrated technology with a microchip. This cannot be achieved by traditional optical analysis owing to the heavy light source and power required. The data show that TES is universal and will accelerate the analytical chemistry toward miniaturized, portable and in situ detection that is challenging with traditional methods and may find applications in the fields of chemistry, biology, environment, and geology, for example, real-time monitoring of ocean pollution, catalytic reaction kinetic, and self-assembly of biological macromolecules.

## Supporting Information Available

Detailed charge calculation, experimental setup, working mechanism of charge induce and transfer, additional contact angle data, data of characteristic TES for ppb level of Fe(NO_3_)_3_, established chemical identification system, detailed data for temperature and humidity effects on the characteristic TES, data for TES on a real sample of tap water with unknown chemical composition, and established chemical analysis database (PDF)
