# Supplementary material for: Triboelectric Spectroscopy for In Situ Chemical Analysis of Liquids
Source: J Am Chem Soc. 2024 Feb 7;146(9):6125–33. doi: 10.1021/jacs.3c13674 (PMC10921404; doi:10.1021/jacs.3c13674)
Supplement: Supplementary file 1 — ja3c13674_si_001.pdf [file ja3c13674_si_001.pdf]

Supporting Information for

Triboelectric spectroscopy for in-situ chemical analysis of liquids

Jinyang Zhang,[△] Xuejiao Wang,[△] Long Zhang, Shiquan Lin,[*] Simone Ciampi,[*] and Zhong Lin Wang[*]

Dr. J. Zhang, X. Wang, Prof. S. Lin, Prof. Z. L. Wang

Beijing Institute of Nanoenergy and Nanosystems

Chinese Academy of Sciences

Beijing 100083, China

E-mail: zlwang@gatech.edu; simone.ciampi@curtin.edu.au; linshiquan@binn.cas.cn

Dr. J. Zhang, Prof. S. Lin, Prof. Z. L. Wang

School of Nanoscience and Technology

University of Chinese Academy of Sciences

Beijing 100049, China

X. Wang

Center on Nanoenergy Research

School of Physical Science and Technology

Guangxi University

Nanning, Guangxi 530004, China

Prof. L. Zhang

Institute of Quantum and Sustainable Technology (IQST),

School of Chemistry and Chemical Engineering,

Jiangsu University
Zhenjiang 212013, China

Prof. S. Ciampi
School of Molecular and Life Sciences
Curtin Institute of Functional Molecules and Interfaces
Curtin University
Bentley, Western Australia 6102, Australia

Prof. Z. L. Wang
School of Materials Science and Engineering
Georgia Institute of Technology,
Atlanta, GA 30332−0245, USA
Yonsei Frontier Lab,
Yonsei University,
Seoul 03722, Republic of Korea

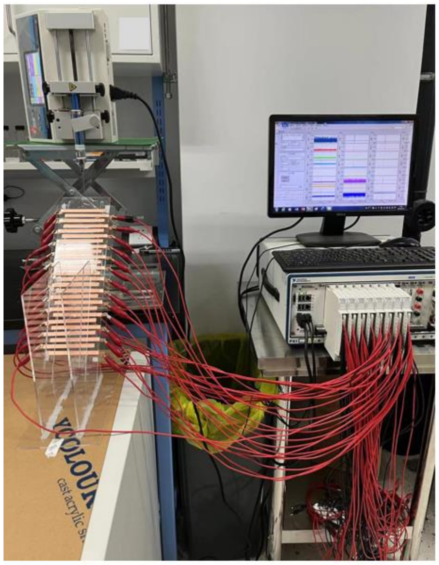

Figure S1. Experimental set-up for triboelectric spectroscopy (TES).

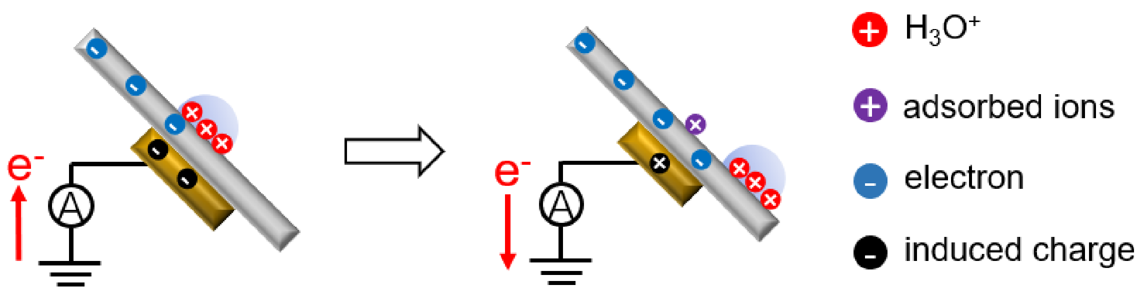

Figure S2. The working mechanism of charge induced at each electrode. When the water droplet is sliding on the FEP film, the electrons will flow from water droplet to FEP film (because FEP has a strong affinity to electrons), with the result being a positively charged water droplet and a negatively charged FEP film, while the positively charged water was started from losing an electron and becomes a cationic hole (H_2O^+) in a quite short lifetime (less than 50 fs). Then the H_2O^+ joins with a neighboring water molecule to yield an OH radical and H_3O^+, according to the chemical reaction of the ionization of liquid water: $H_2O^+ + H_2O \rightarrow OH + H_3O^+$.[1] When a droplet touching the FEP film with the copper electrode beneath it, an induced current was detected, after the current peak is integrated, the corresponding transferred charge Qc was calculated. Similarly, we can get the corresponding transferred charge Qs when the droplet separated from the FEP surface with the copper electrode beneath it. The difference between Qc and Qs ($||Qs| - |Qc||$) indicate the transferred charge on a single electrode. The induced charge can be shielded when ions adsorbed on the FEP surface after drop moves away.

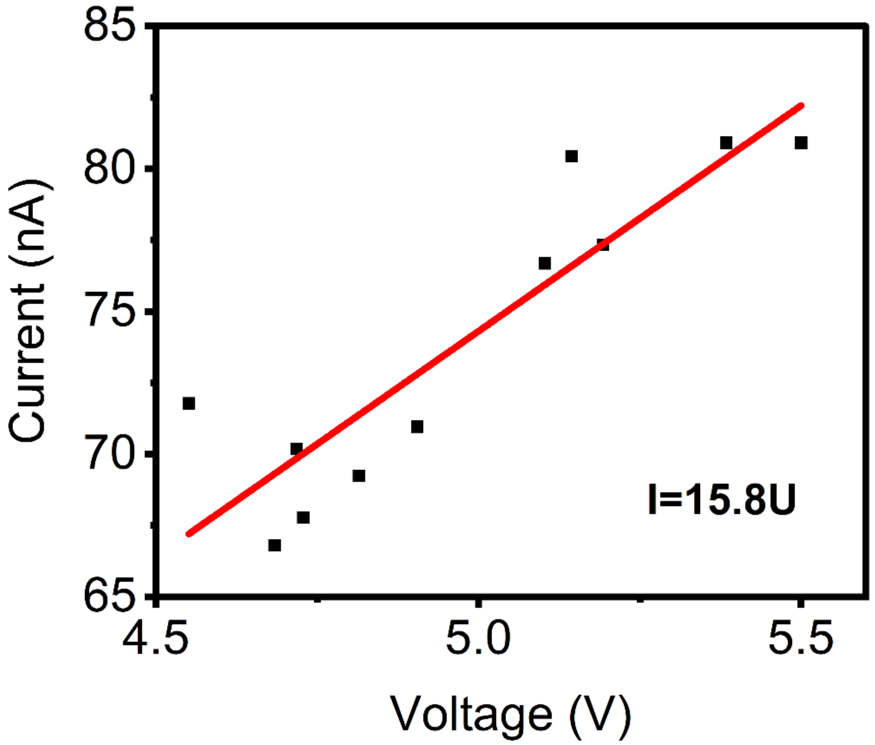

Figure S3. Calculation of the transferred surface charge at each electrode. In our experiments, the transferred charge on the FEP surfaces needs to be calculated as we measured is the voltage generated by contact electrification (CE) between liquid and FEP. First, we need to convert the voltage to current value. It is known that the voltage and current are linear relationship if the resistance is constant, thus we measured the voltage and current for different electrodes at the same time by NI PXle-8880 electrometer and Keithley 6514 electrometer. And the relationship between the voltage and current was obtained. After that we can get the corresponding transferred charge by integrating the obtained current curve, this is same with our published work.[2]

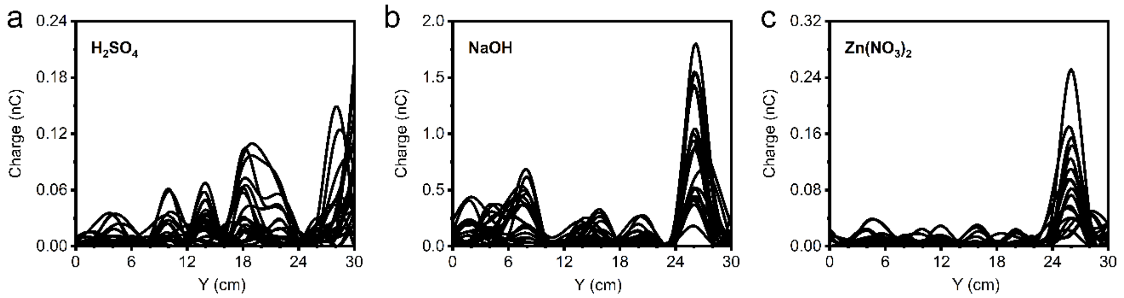

Figure S4. 20 independent spectroscopies for H_2SO_4 (pH 3), NaOH (pH 13) and $Zn(NO_3)_2$ (1M).

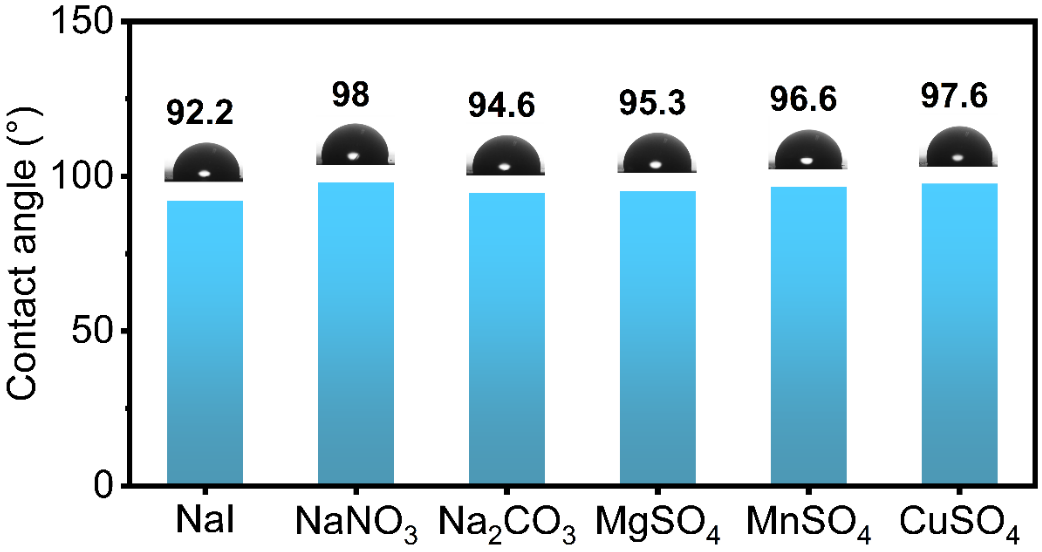

Figure S5. Contact angles of NaI, $NaNO_3$, Na_2CO_3, $MgSO_4$, $MnSO_4$ and $CuSO_4$ droplets on the FEP film.

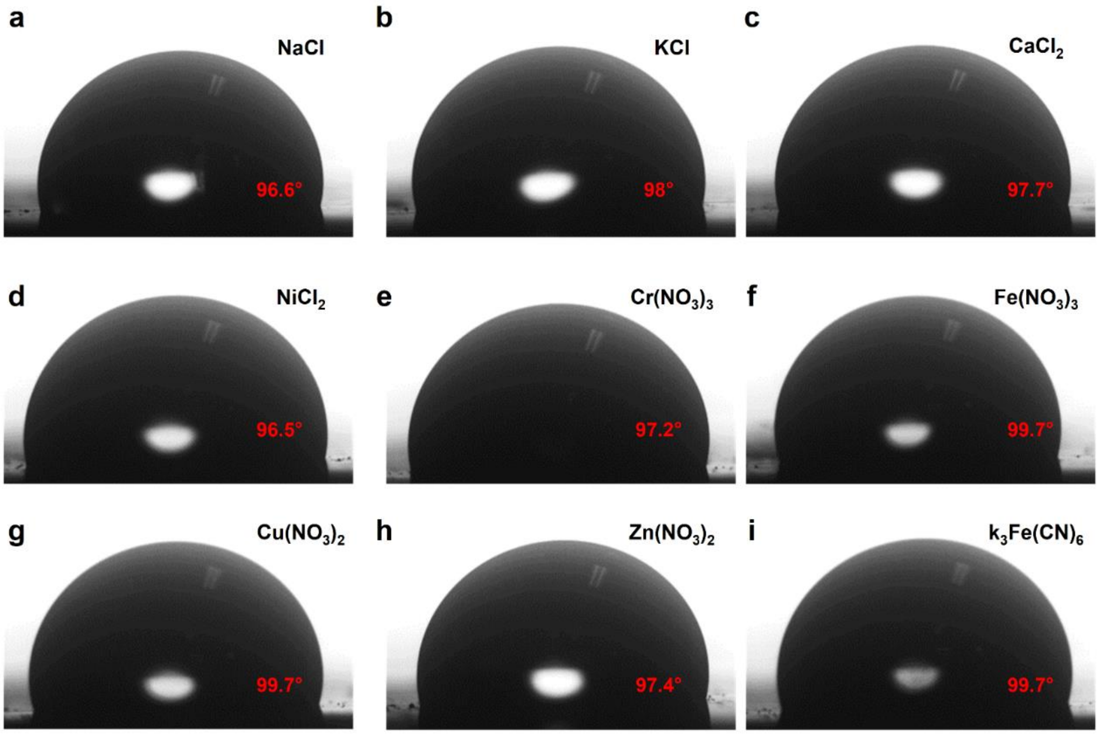

Figure S6. Measurements of static water contact angles for different liquid samples on FEP surface.

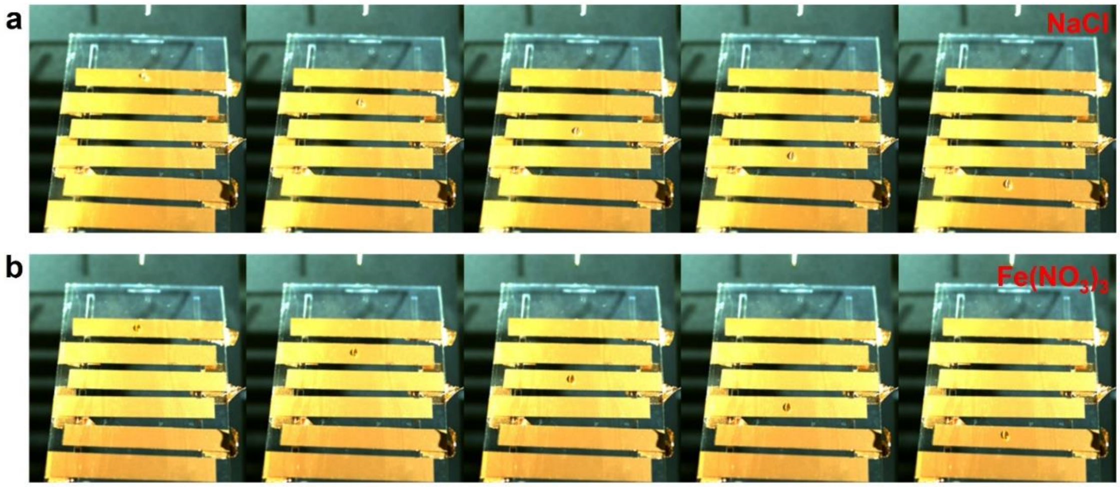

Figure S7. Snapshots of droplets with different chemicals sliding on FEP surface.

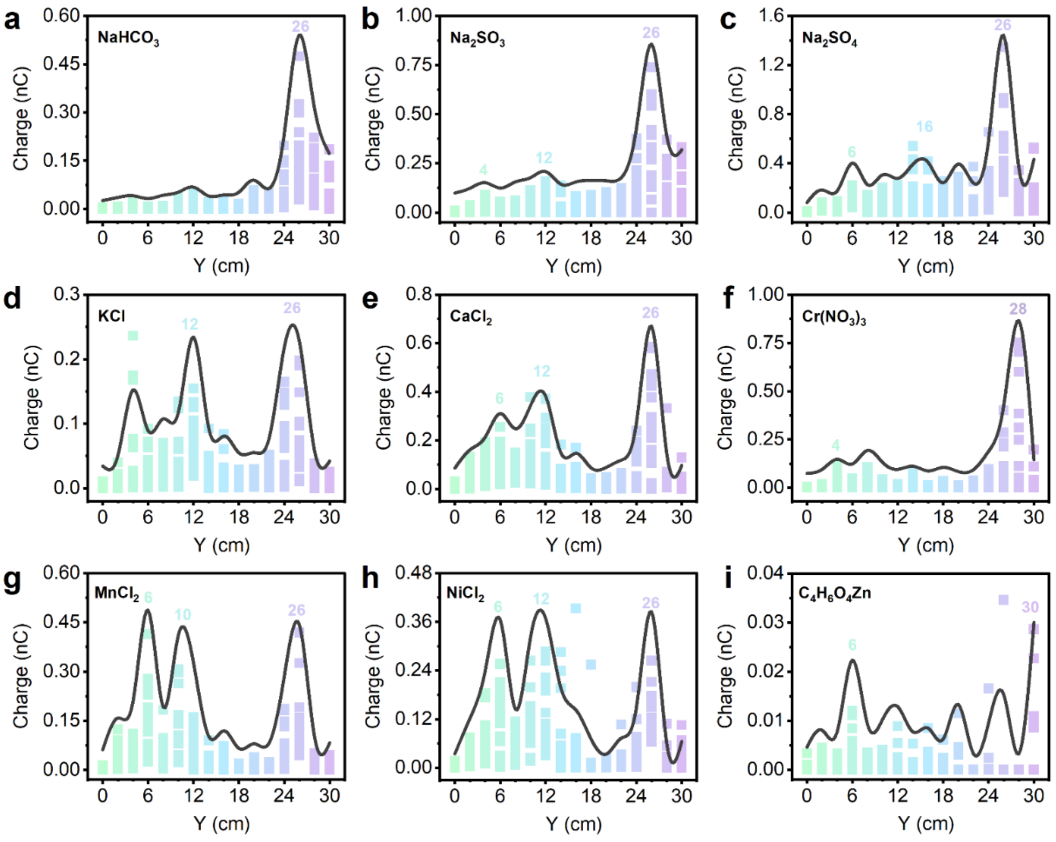

Figure S8. TES recorded for various chemicals.

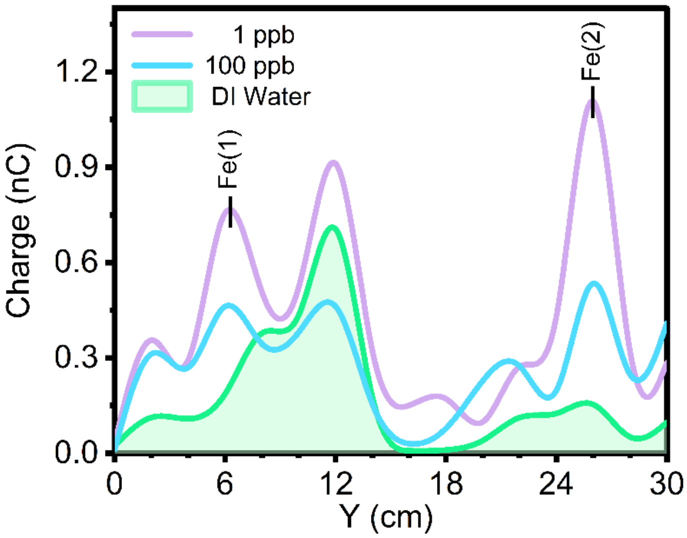

Figure S9. Performance of characteristic TES for ppb level of $Fe(NO_3)_3$. When 1 and 100 ppb of $Fe(NO_3)_3$ was added to DI water, obvious Fe^{3+} peaks appeared in the spectroscopy compared with pure DI water. The high Fe^{3+} concentration in the sample will inhibit the interface charge transfer, which is probably due to the excessive free ions in the droplets can interfere with the electron-transfer process because of the screen effect.[3-4]

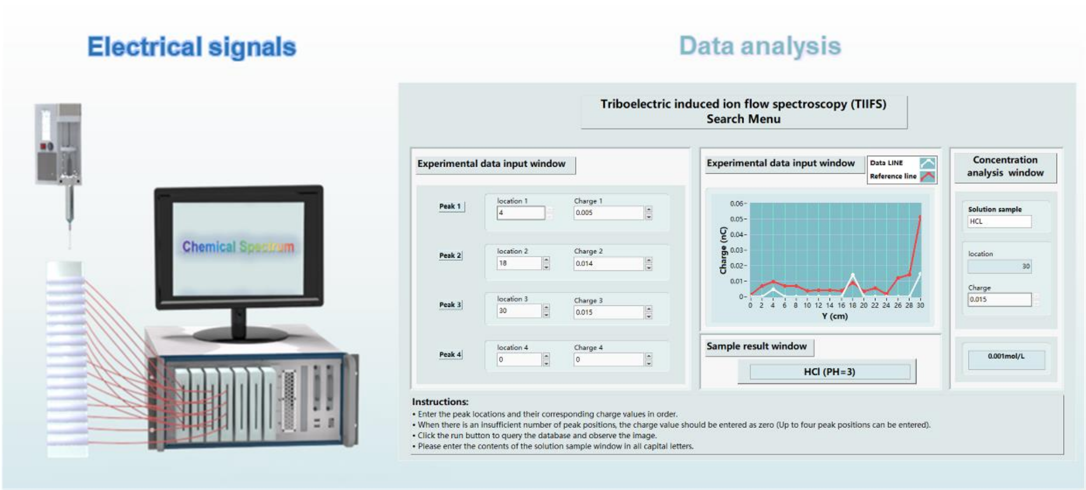

Figure S10. The experimental setup shows the combination of data extraction and chemical identification system.

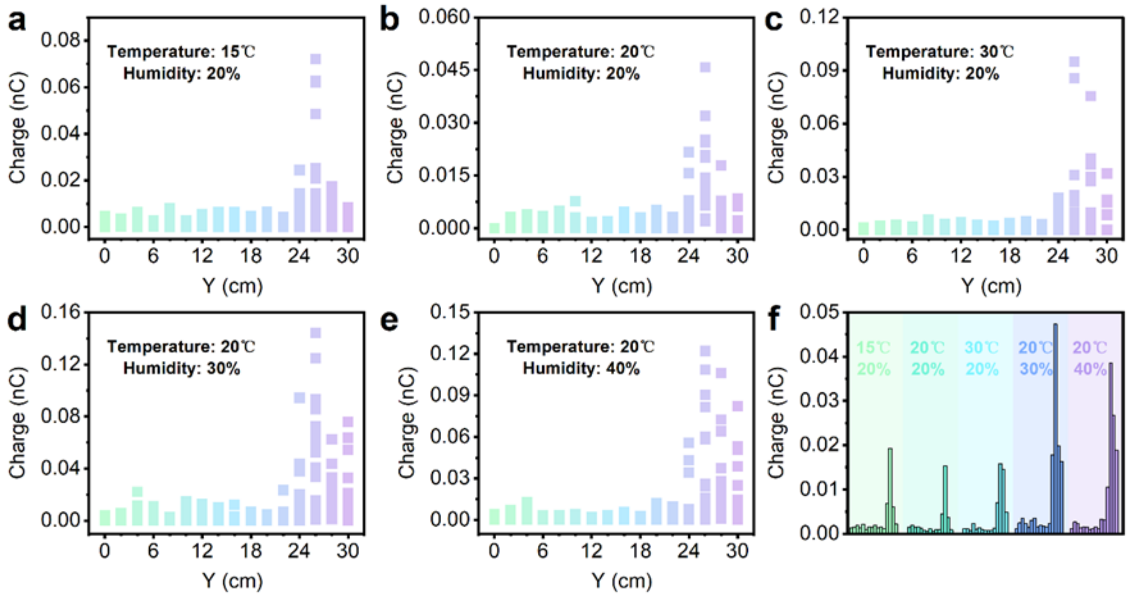

Figure S11. **Temperature and humidity effects on the characteristic TES.** (a-c) Characteristic TES of 1M $Cu(NO_3)_2$ at different temperatures (humidity keep as 20%). (d-e) Characteristic TES of 1M $Cu(NO_3)_2$ at different humidity (temperature keep as 20°), the small squares are the data of 20 independent experiments. (g) Compared the samples measured at different temperatures and humidities. The data represents the average values of 20 independent experiments.

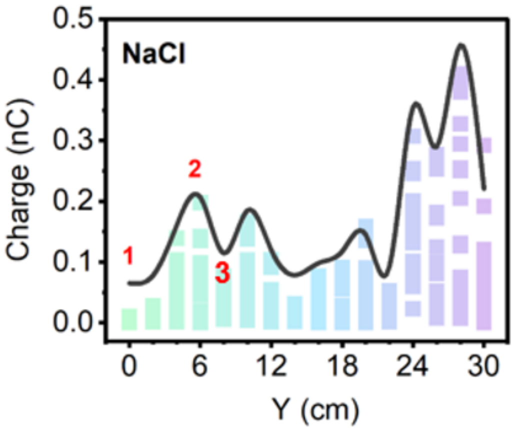

Figure S12. Characteristic TES recorded for 1M NaCl. Position 1 represents when the droplet just comes into contact-electrification with FEP, position 2 is the first peak position of NaCl, while position 3 is after the peak.

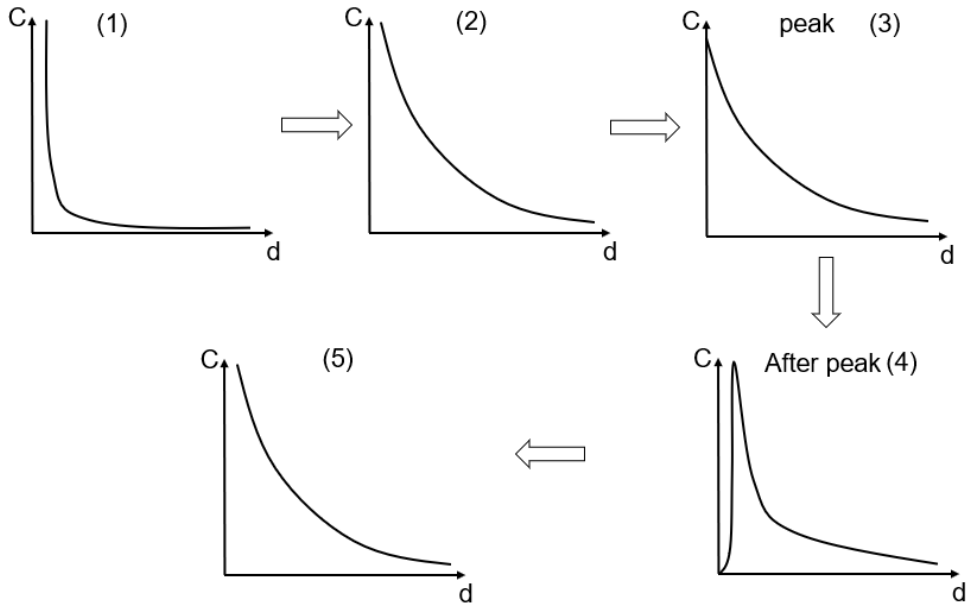

Figure S13. Schematic illustration of the continuous change of the interfacial concentration distribution of H_3O^+ at liquid-solid interface. When H_3O^+ are first generated at the interface, the concentration of H_3O^+ is high (1), and they begin to diffuse to the liquid droplets. When the diffusion reaches a certain level (2), the concentration gradient of H_3O^+ at the interface decreases, and the H_3O^+ started to incline to adsorb on FEP surface due to electrostatic attraction. While, the number of shielded H_3O^+ increases, the electrostatic attraction of electrons to Na^+ begin to decrease. At a certain moment, the adsorption of H_3O^+ on the surface of FEP reaches the maximum (3). In this way, the situation is very similar to FEP contacts with pure water (the contact electrification between water and FEP has the highest charge transfer),[3-4] and when the droplet slides away, most of adsorbed H_3O^+ be taken away by moving droplet, thus the appearance of peaks observed. After peak, concentration of H_3O^+ at droplet boundary is higher than the FEP surface (4), so the H_3O^+ start to adsorb towards the FEP until another equilibrium is reached (5). Here, c represents the concentration of H_3O^+ at liquid-solid interface, while d represents the distance from the solid surface.

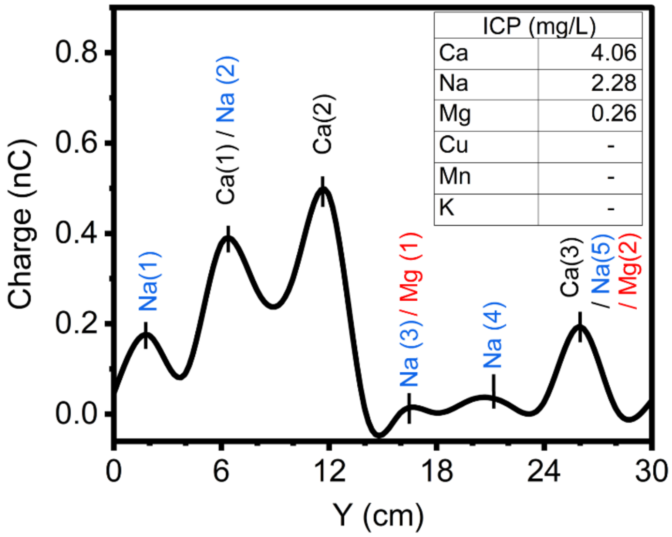

Figure S14. Performance of characteristic TES on a real sample of tap water with unknow chemical composition. When a real tap water droplet slides through FEP surface, the spectroscopy shows a high probability of Ca^{2+}, Na^{+}, and Mg^{2+} in the sample compared to our database, which is consistent with ICP measurement (inserted in the Figure). Moreover, there is most likely to be SO_4^{2-} in the tap water sample as the characteristic spectrum for Na^{+} is consistent with Na_2SO_4. The number of independent tests for this sample is 5.

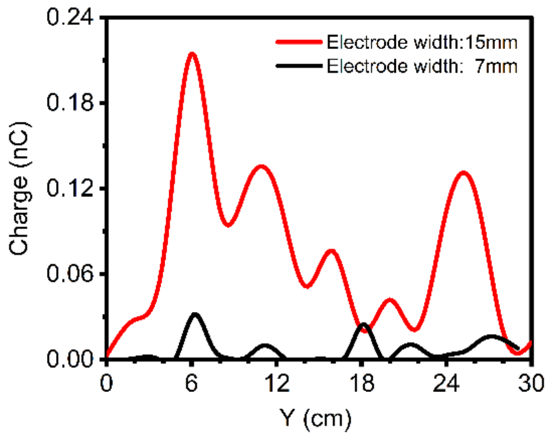

Figure S15. Influence of the arrangement and size of the electrode array on the spectroscopy of 1M NaCl.

Table S1. The chemical analysis database

Samples	Peak location and Q(nc)					Peak-to-peak ratio	Diagram
	First	Second	Third	Fourth	Fifth		
NaCl	6<br>0.07 ± 0.05	10<br>0.06 ± 0.05	24<br>0.14 ± 0.07	28<br>0.18 ± 0.15	-	1 : 1.1 : 2.3 : 3	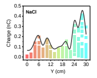
NaI	8<br>0.003 ± 0.001	12<br>0.003 ± 0.004	26<br>0.036 ± 0.024	-	-	1 : 1 : 12	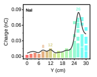
$NaNO_3$	4<br>0.02 ± 0.03	12<br>0.03 ± 0.03	26<br>0.10 ± 0.07	-	-	1 : 1.5 : 5	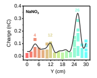
$NaHCO_3$	12<br>0.01 ± 0.01	20<br>0.02 ± 0.02	26<br>0.16 ± 0.11	-	-	1 : 2 : 16	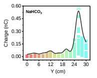
Na_2CO_3	6<br>0.05 ± 0.04	10<br>0.04 ± 0.04	24<br>0.20 ± 0.17	-	-	1.25 : 1 :5	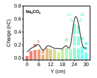
Na_2SO_3	4<br>0.02 ± 0.03	12<br>0.05 ± 0.05	26<br>0.32 ± 0.21	-	-	1 : 2.5 : 16	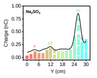
Na_2SO_4	2<br>0.04± 0.02	6<br>0.12 ± 0.05	16<br>0.12 ± 0.13	20<br>0.11 ± 0.11	26<br>0.47 ± 0.30	1: 3 : 3: 2.75:11.75	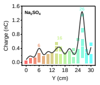
$MgSO_4$	16<br>0.36 ± 0.25	26<br>1.89 ± 1.32	-	-	-	1: 5.25	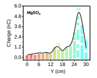
KCl	4<br>0.04 ± 0.07	12<br>0.07 ± 0.04	26<br>0.07 ± 0.05	-	-	1 : 1.75 : 1.75	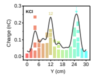
$K_3Fe(CN)_6$	4<br>0.02 ± 0.02	26<br>0.16 ± 0.06		-	-	1 : 8	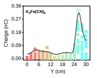
$CaCl_2$-$2H_2O$	6<br>0.12 ± 0.06	12<br>0.15 ± 0.09	26<br>0.28 ± 0.17	-	-	1 : 1.25 : 2.33	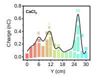
$Cr(NO_3)_3$-$9H_2O$	4<br>0.02 ± 0.03	8<br>0.03 ± 0.04	28<br>0.21 ± 0.27	-	-	1 : 1.5 : 10.5	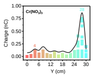
$MnCl_2$-$4H_2O$	6<br>0.14 ± 0.11	10<br>0.11 ± 0.10	26<br>0.13 ± 0.10	-	-	1.27 : 1 : 1.18	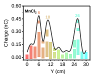
$MnSO_4$-H_2O	14<br>0.73 ± 0.65	24<br>0.79 ± 0.52	28<br>0.89 ± 0.61	-	-	1 : 1.08 : 1.22	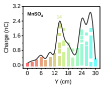
$Fe(NO_3)_3$-$9H_2O$	6<br>4.3×10^{-4} ± 6.5×10^{-4}	16<br>0.005 ± 0.009	28<br>0.04 ± 0.04	-	-	1 : 11 : 93	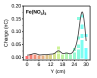
$NiCl_2$-$6H_2O$	6<br>0.13 ± 0.07	12<br>0.13 ± 0.08	26<br>0.13 ± 0.05	-	-	1 : 1 : 1	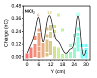
$CuSO_4$	10<br>0.09 ± 0.09	14<br>0.14 ± 0.17	24<br>0.09 ± 0.11	28<br>0.08 ± 0.11	-	1.3 : 1.75 : 1.3 : 1	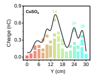
$Cu(NO_3)_2$-$3H_2O$	26<br>0.02 ± 0.01	-	-	-	-	-	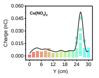
$Zn(NO_3)_2$-$6H_2O$	4<br>0.01 ± 0.01	26<br>0.09 ± 0.06	-	-	-	1 : 9	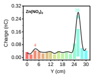
$C_4H_6O_4Zn$	6<br>0.004 ± 0.004	20<br>0.002 ± 0.003	26<br>0.002 ± 0.009	30<br>0.005 ± 0.009	-	2 : 1 : 1 : 2.5	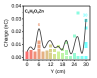

HCl<br>(pH=5)	4<br>0.98 ±<br>0.35	20<br>1.15 ±<br>0.45	30<br>1.65 ±<br>0.47	-	-	1 : 1.17 : 1.68	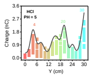
HCl<br>(pH=4)	4<br>0.45 ±<br>0.15	8<br>0.70 ±<br>0.27	20<br>0.66 ±<br>0.25	30<br>0.91 ±<br>0.31	-	1 : 1.56 : 1.44 : 2.02	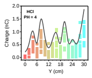
HCl<br>(pH=3)	4<br>0.01 ±<br>0.01	18<br>0.01 ±<br>0.01	30<br>0.05 ±<br>0.05	-	-	1 : 1 : 5	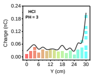
H_2SO_4<br>(pH=3)	10<br>0.02 ±<br>0.02	14<br>0.03 ±<br>0.02	18<br>0.04 ±<br>0.03	30<br>0.07 ±<br>0.05	-	1 : 1.5 : 2 : 3.5	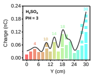
HNO_3<br>(pH=3)	4<br>0.07 ±<br>0.04	14<br>0.10 ±<br>0.09	18<br>0.07 ±<br>0.08	30<br>0.12 ±<br>0.14	-	1 : 1.43 : 1 : 1.71	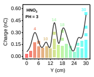
KOH<br>(pH=9)	4<br>1.06 ±<br>0.57	8<br>1.00 ±<br>0.48	14<br>0.52 ±<br>0.26	26<br>2.62 ±<br>0.77	-	2.04 : 1.92 : 1 : 5.04	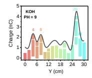
KOH<br>(pH=11)	4<br>1.16 ±<br>0.42	8<br>0.91 ±<br>0.48	14<br>0.54 ±<br>0.24	26<br>2.41 ±<br>0.59	-	2.15 : 1.69 : 1 : 4.46	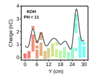
KOH<br>(pH=13)	4<br>0.40 ±<br>0.17	8<br>0.17 ±<br>0.11	16<br>0.15 ±<br>0.11	26<br>1.09 ±<br>0.39	-	2.67 : 1.13 : 1 : 7.27	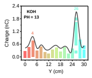
NaOH<br>(pH=13)	8<br>0.29 ±<br>0.19	16<br>0.10 ±<br>0.10	26<br>0.86 ±<br>0.47	-	-	2.9 : 1 : 8.6	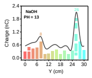
CH_3CH_2OH	4<br>1.8×10^{-4} ±<br>4.3×10^{-4}	28<br>0.03 ±<br>0.01	-	-	-	1: 166.7	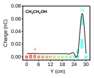
C_3H_6O	14<br>0.002 ±<br>0.002	28<br>0.04 ±<br>0.02	-	-	-	1 : 20	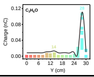

Reference:

1. Loh, Z.-H.; Doumy, G.; Arnold, C.; Kjellsson, L.; Southworth, S.; Al Haddad, A.; Kumagai, Y.; Tu, M.-F.; Ho, P.; March, A., Observation of the Fastest Chemical Processes in the Radiolysis of Water. *Science* **2020,** *367* (6474), 179-182.
2. Zhang, J.; Lin, S.; Wang, Z. L., Triboelectric Nanogenerator Array as a Probe for In Situ Dynamic Mapping of Interface Charge Transfer at a Liquid–Solid Contacting. *ACS Nano* **2023,** *17* (2), 1646-1652.
3. Zhang, J.; Lin, S.; Zheng, M.; Wang, Z. L., Triboelectric Nanogenerator as a Probe for Measuring the Charge Transfer between Liquid and Solid Surfaces. *ACS Nano* **2021,** *15* (9), 14830-14837.
4. Zhan, F.; Wang, A. C.; Xu, L.; Lin, S.; Shao, J.; Chen, X.; Wang, Z. L., Electron Transfer as a Liquid Droplet Contacting a Polymer Surface. *ACS Nano* **2020,** *14* (12), 17565-17573.
